# Pigeon pea crop stage strongly influences plant susceptibility to *Helicoverpa armigera* (Lepidoptera: Noctuidae)

**DOI:** 10.1093/jee/toae050

**Published:** 2024-04-02

**Authors:** Trevor M Volp, Myron P Zalucki, Michael J Furlong

**Affiliations:** Agri-Science Queensland, Department of Agriculture and Fisheries, Toowoomba, QLD 4350, Australia; School of the Environment, The University of Queensland, St Lucia, QLD 4072, Australia; School of the Environment, The University of Queensland, St Lucia, QLD 4072, Australia; School of the Environment, The University of Queensland, St Lucia, QLD 4072, Australia

**Keywords:** phenology, host-plant resistance, herbivory, preference–performance hypothesis

## Abstract

*Helicoverpa armigera* Hübner (Lepidoptera: Noctuidae; Hübner) is the major insect pest of pigeon pea [*Cajanus cajan; Fabales: Fabaceae;* (L.) Millspaugh] worldwide. Research to develop pest management strategies for *H. armigera* in pigeon pea has focused heavily on developing less susceptible cultivars, with limited practical success. We examined how pigeon pea crop stage influences plant susceptibility to *H. armigera* using a combination of glasshouse and laboratory experiments. Plant phenology significantly affected oviposition with moths laying more eggs on flowering and podding plants but only a few on vegetative plants. Larval survival was greatest on flowering and vegetative plants, wherein larvae mostly chose to feed inside flowers on flowering plants and on the adaxial surface of expanding leaves on vegetative plants. Larval survival was poor on podding plants despite moths laying many eggs on plants of this stage. When left to feed without restriction on plants for 7 days, larvae feeding on flowering plants were >10 times the weight of larvae feeding on plants of other phenological stages. On whole plants, unrestricted larvae preferred to feed on pigeon pea flowers and on expanding leaves, but in no-choice Petri dish assays *H. armigera* larvae could feed and survive on all pigeon pea reproductive structures. Our results show that crop stage and the availability of flowers strongly influence pigeon pea susceptibility to *H. armigera*. An increased understanding of *H. armigera*-pigeon pea ecology will be useful in guiding the development of resistant varieties and other management tactics.

## Introduction

Understanding insect–plant interactions in pest-crop systems is a prerequisite to developing crop cultivars and/or cropping systems that suppress insect pest populations and avoiding dependence on synthetic insecticides. The central tenet that guides the study of insect–plant interactions is the preference–performance hypothesis (PPH), which posits that adult herbivores should oviposit eggs on plants or on plant parts that maximize larval performance (Mayhew 2001).

Although there is substantial empirical support for the PPH (Gripenberg et al. 2010, Jones 2022), there are also experimental studies that fail to support it (Jallow and Zalucki 2003, Cotter and Edwards 2006, Gripenberg et al. 2010, Silva and Furlong 2012, Jones 2022). There are several reasons why the PPH may not be supported in a specific insect–plant system including host range of the herbivore, larval or adult experience with another plant species, different requirements between insect life stages, lack of cues to guide adult selection, plant “quality” varying with space and time, and the ability of mobile immature stages to move away from unsuitable feeding sites where eggs were laid (Potter et al. 2012, Ang et al. 2014, Gómez Jiménez et al. 2014).

In this study, we examine the preference and performance of the polyphagous pest *Helicoverpa armigera* (Lepidoptera: Noctuidae) on the major pulse crop pigeon pea [*Cajanus cajan* (L.) Millspaugh]. *Helicoverpa armigera* is geographically widespread, feeds on numerous crop species (Cunningham and Zalucki 2014), causes substantial yield losses resulting in significant management costs, and evolves resistance to insecticides (Fitt 1989, Walsh et al. 2022). Moths are highly attracted to the flowering stages of host plants and larvae tend to feed on plant reproductive structures (Liu et al. 2010). The feeding preference for reproductive structures (i.e., the yield-forming plant organs) explains, in part, why *H. armigera* infestations can cause considerable crop yield loss to a wide range of hosts (Zalucki et al. 1986).

Pigeon pea is grown throughout the semiarid tropics and subtropics and is a source of food and income for some of the world’s poorest people (Mula and Saxena 2010). *Helicoverpa armigera* is the major biotic constraint to global pigeon pea production, and its management relies heavily on the application of insecticides (Shanower et al. 1999, Sharma et al. 2012). Although a large body of research has attempted to develop *H. armigera*-resistant pigeon pea cultivars, there has not been practical success (Sharma 2016). Arguably, host-plant resistance research in pigeon pea has suffered from 2 main limitations: trade-offs between plant resistance traits and agronomic suitability and a limited understanding of the insect–plant interactions of the study system (Volp et al. 2023).

Before varieties that are less susceptible to *H. armigera* can be developed, the basis of pigeon pea susceptibility to *H. armigera* and details of the specific insect–plant interactions involved need to be properly understood. Moths are attracted to pigeon pea volatiles (Rajapakse et al. 2006), and eggs are then mostly oviposited on floral structures, sites wherein larvae establish (Rajapakse and Walter 2007, Volp et al. 2023). As larvae develop, they are purported to “switch” to feeding on pods, where they can cause substantial yield loss (Green et al. 2002, Rajapakse 2007). Flowers likely play an important role in pigeon pea susceptibility to *H. armigera*, but the influence of the pigeon pea plant stage on oviposition and establishment of this species is not fully understood.

In this study, we investigate how pigeon pea crop stage influences plant susceptibility to *H. armigera*. We examine moth oviposition preference for different crop stages, oviposition site selection at different crop stages, a larval establishment when restricted to different oviposition sites (at different crop stages), larval establishment and early instar performance on different crop stages when unrestricted (i.e., allowed to “choose” sites), and larval survival on different pigeon pea reproductive structures.

## Materials and Methods

### Plants

Pigeon pea plants of a short-duration, determinate, *H. armigera*-susceptible cultivar (ICPL 86012) were grown in a controlled temperature glasshouse (27 °C day, 25 °C night) under natural photoperiod at the Queensland Department of Agriculture and Fisheries site in Toowoomba, Australia (−27.534137, 151.929201). Pigeon pea seeds were planted in 200 mm (4L) ANOVA pots using a 2:1 mix of commercial potting mix (Searles Premium) and sand. Plants were watered as required, and no additional fertilizer was provided. No insecticides were applied to the plants, but they were regularly inspected for any glasshouse pests, which were physically removed upon detection. For all experiments, we conducted weekly plantings to enable simultaneous comparisons of different plant stages.

Reproductive development in pigeon peas occurs as follows: racemes form with bud initials, which develop into buds that then open to flowers. Flowers remain open for several days, and when they are fertilized, the ovary begins to develop, and a pod forms. The flower petals then desiccate as the pod continues to expand. Once pods are fully expanded, seeds commence filling with assimilates, and when they are filled, the pods start to harden and dry down. For simplification of this continuous process, we have split pigeon pea reproductive structures into 7 categories (Table 1), the terms of which are used throughout this article. During pigeon pea reproductive stages, these structures overlap in their availability, particularly for indeterminate cultivars. The duration of these structures on plants differs based on genotype and environment (Mahendraraj 2022); however, some approximate guidelines are as follows: bud initial development and conversion to an open flower takes 1–2 wk, flowers remain open for <1 day to several days, pods expand over 1–2 wk, and seed development and maturation takes approximately 3 wk (Reddy 1990, Mahendraraj 2022, Volp et al. unpublished data).

**Table 1.** Reproductive structures/stages present on pigeon pea plants during reproductive development

Structure	Details
Bud initial	Calyx encloses petals
Bud	Petals expanded out of calyx but not open
Flower	Petals open
Spent flower	Flower fertilized and commenced desiccating
Small expanding pod	Pod expanding, <15 mm, smaller than the size of a flower (therefore often hidden inside spent flower petals)
Large expanding pod	Pod expanding, 15–50 mm, not filling seeds
Filling pod	Fully expanded, filling seeds

### Insects

*Helicoverpa armigera* moths and larvae were obtained from a laboratory culture maintained in a controlled temperature room (25 ± 2 °C; L: D 12:12) at the Queensland Department of Agriculture and Fisheries laboratory in Toowoomba. The culture was established in 2020, and insects were collected from various field crops in South-East Queensland, Australia, with specimens added regularly to minimize inbreeding. Adult moths were kept in 5-L plastic buckets and supplied with 10% sucrose solution using a cotton wick in 70 ml plastic containers. An 18 cm hole was cut in the bucket lid, and the edges of the lid were used to secure the nappy liner (bamboo rayon), which was used as an oviposition substrate. Eggs were removed daily, washed in 1% sodium hypochlorite solution, rinsed with distilled water, and collected onto filter paper using vacuum filtration. The filter paper was allowed to air dry and then placed in Petri dishes (90 mm diameter), which were sealed with Parafilm and incubated until neonates hatched. Upon hatching, neonate larvae were placed in 500 ml plastic containers and were maintained on a soybean flour-based artificial diet (recipe modified from Teakle and Jensen 1985, see Volp et al. 2023; Supplementary Table S1 for ingredient list). Upon reaching the third instar, larvae were transferred to a fresh diet in 32-well plastic trays (12 ml per well), where they remained until pupation. Newly formed pupae were washed in 1% sodium hypochlorite, rinsed with distilled water, air-dried, and placed in 500 ml plastic containers until eclosion.

### Oviposition No-Choice

We examined the oviposition behavior of *H. armigera* at 3 crop stages: vegetative (5 wk postplanting), flowering (8 wk), and podding (11 wk). The vegetative plants had no reproductive structures, the flowering plants had mostly floral structures, and the podding plants had stopped producing new flowers and were filling pods (Supplementary Table S1). Newly emerged moths (<24 h post eclosion) were obtained from the laboratory colony and placed in groups of 3 males and 3 females in oviposition cages (69 cm × 69 cm × 122 cm) in the glasshouse. Three plants of the appropriate phenological stage were placed in an equilateral triangle (8 cm distance between pots) in the oviposition cages. A single cage with 3 plants of the given phenological stage constituted a replicate, and 3 cages (i.e., one for each crop stage treatment) were run simultaneously. Each treatment was replicated 11 times.

Moths were provided access to 10% sugar solution through a wick in 70mL plastic containers, to ensure all treatments had access to a carbohydrate food supply. Moths were allowed to mate and oviposit for 4 nights, after which time they were removed, and the plants were searched for eggs. We recorded the number and location of each egg on each plant together with plant morphometric variables (height, number of nodes, and the number of different plant structures present). After the first 6 replicates, we noticed the strong effect of crop stage on egg counts. To examine if crop stage was influencing moth mating behavior, we retained female moths from the final 5 replicates for dissection and enumeration of spermatophores. We counted spermatophores by dissecting the abdomens of female moths in an ethanol-water solution under a stereomicroscope (Nikon: SMZ800N).

### Caged Early Instar Establishment—3 Days Duration

Based on the results of the no-choice oviposition experiment, we examined the performance of early instar *H. armigera* on different plant parts at different crop stages: leaves and growing tips on vegetative plants; leaves and racemes (i.e., flowers) on flowering plants; and leaves and racemes (i.e., pods) on podding plants. In glasshouse experiments, groups of 5 neonate *H. armigera* (<2 h old) were placed at the respective locations on plants of the appropriate stage. Larvae were restricted to the plant location by placing organza mesh bags (13 cm  × 12 cm) over a single trifoliate leaf in the “leaves” treatment, but in the “raceme” and “vegetative tip” treatments, the bags covered the top 3 nodes. After 3 days, the relevant plant parts were excised and returned to the laboratory, where they were inspected for larvae. Survival and larval instar were recorded as performance measures. For each treatment, replication occurred at the level of a single plant, and each treatment (crop stage × plant location) was replicated 5 times.

### Uncaged Early Instar Establishment—3 Days Duration

We also examined the performance and feeding locations of unrestricted early instar larvae when placed at the same plant location × plant stage combinations as in the caged early instar experiment. The uncaged experiment examined the establishment when larvae could move and self-select feeding sites. As in the caged experiment, groups of 5 neonate larvae (<2 h old) were placed on different plant parts of plants at different crop stages: leaves and growing tips on vegetative plants; leaves and racemes (i.e., flowers) on flowering plants; and leaves and racemes (i.e., pods) on podding plants. After 3 days, plants were searched for larvae. We recorded larval survival, development, and the location of larvae (i.e., plant structure and location within the structure). To account for the typically larger variation resulting from examining unrestricted larvae on whole plants, this experiment was replicated 10 times.

### Unrestricted Larvae on Whole Plants—7 Days Duration

Because *H. armigera* larvae can survive substantial periods (up to 132 h) without feeding (Luong et al. 2018), we conducted a longer-duration experiment examining the performance of unrestricted larvae. We placed neonates on plants as described previously (*n* = 5 per plant). However, in this experiment, we only started neonates at the most common oviposition sites for each crop stage: expanded leaves at the vegetative stage, flowers at flowering, and pods at podding. At 7 days, we destructively harvested plants and recorded larval survival, development, weight, and location. Treatments were replicated 5 times.

### Larval Survival on Plant Reproductive Structures—4 Days Duration

We examined the ability of *H. armigera* larvae to feed and survive when restricted to different pigeon pea reproductive structures (Table 1) in a no-choice Petri dish assay. This experiment investigated if early instar larvae are unable to feed on certain plant structures or if they likely move to preferred feeding sites. We investigated larvae feeding on bud initials, buds, flowers, and small expanding pods (<15 mm) taken from flowering plants; large expanding pods (>15 mm) taken from late flowering-early podding plants; and large filling pods taken from podding plants.

We placed a single neonate in a Petri dish (90 mm diameter) and provided access to a single structure that was placed on filter paper moistened with distilled water. We included a starvation treatment, where larvae were not provided with any plant parts as food sources. The experiment was conducted in a controlled temperature room (25 °C ± 2, L: D 12:12). We monitored larval survival every 24 h and terminated the experiment after 4 days. For each treatment, a single larva and the given plant part combination in a single Petri dish were replicated, and we conducted 30 replicates per treatment.

### Statistical Analysis

All experiments were randomized block designs. Response variables for most experiments were analyzed with ANOVAs, using replicate as a blocking factor. Fisher’s LSD test was used for post hoc comparisons. Larval distribution data from the unrestricted larval experiments were analyzed by calculating the proportion of live larvae located at a plant location for each replicate, then analyzing the proportional data with ANOVA using plant location as the independent variable. Kruskal–Wallis tests were used to analyze larval distribution for the flowering and podding treatments, as data did not meet the assumptions required for ANOVA. A chi-square test was used to analyze larval survival in the plant part feeding experiment in Petri dishes. All analyses were performed in R version 3.6.2 (R Core Team 2019); for Fisher’s LSD tests, we used the package “*agricolae*” (de Mendiburu 2020), and graphs were made with the package “*ggplot2*” (Wickham et al. 2016).

## Results

### Oviposition No-Choice

**Fig. 1.**
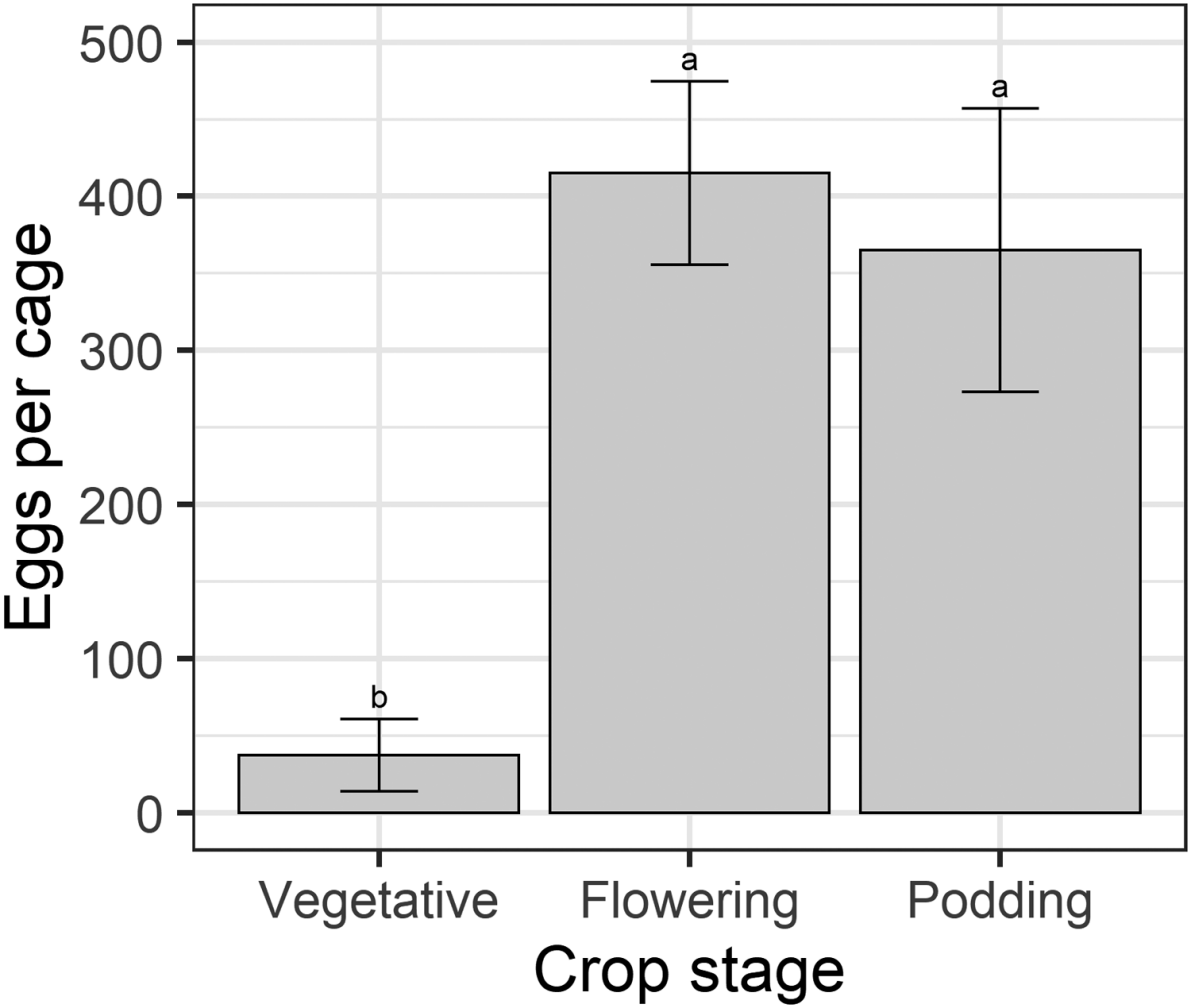
Mean total eggs laid on plants (3 plants per cage) of different crop phenological stages from the oviposition no-choice experiment. Bars represent the means, and the error bars represent standard errors. Different letters indicate a difference according to Fisher’s LSD test.

Crop stage had a significant effect on how many eggs were laid on plants, and more eggs were laid on flowering and podding plants compared to vegetative plants (*F* = 8.95; *df* = 2, 25; *P* = 0.0017; Fig. 1). The numbers of eggs laid by *H. armigera* moths were highly variable. There were 2 cages where no eggs were laid at all (both containing vegetative plants), whereas the maximum number of eggs laid in a cage was 867 (equating to 289 eggs per female moth).

**Fig. 2.**
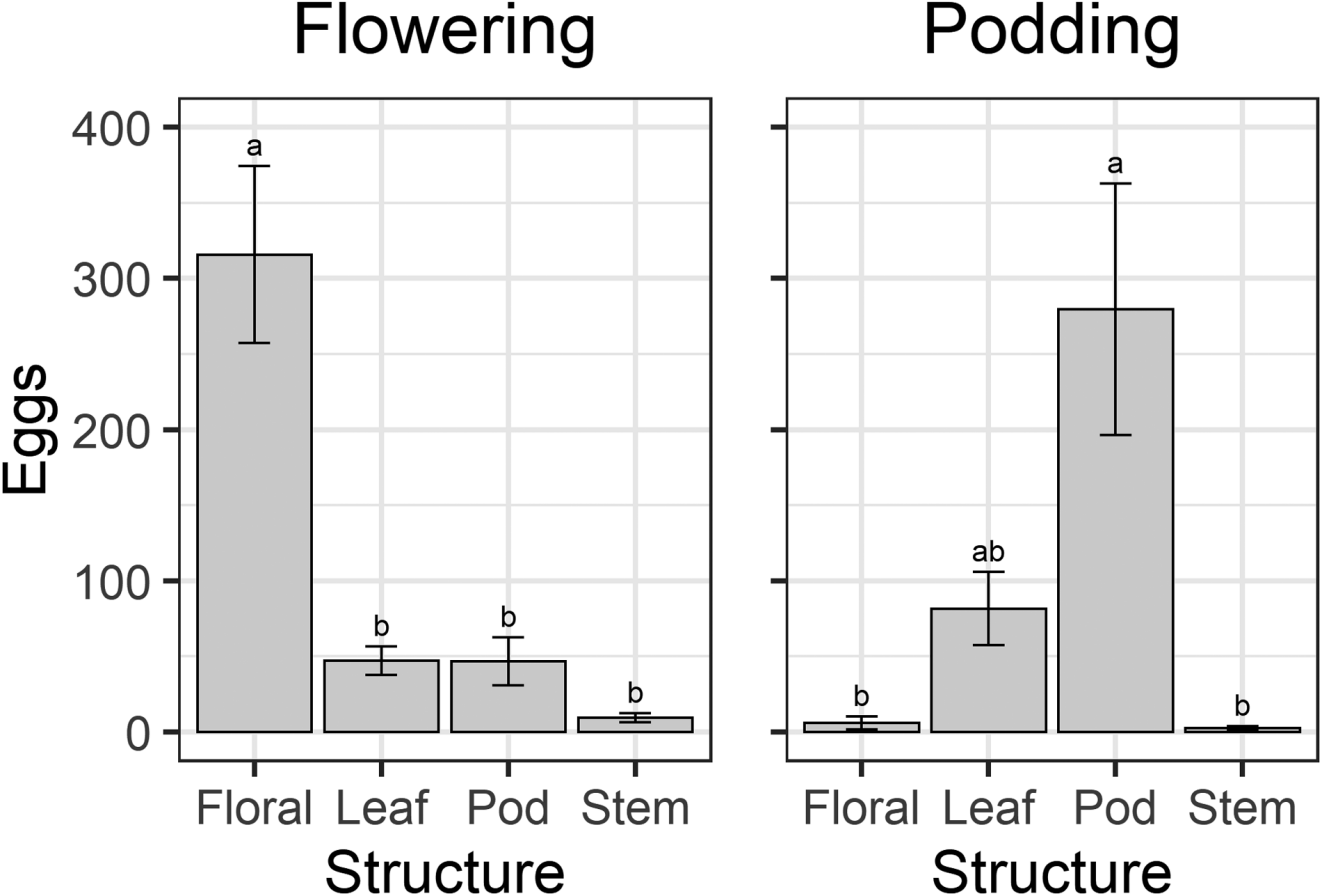
Mean number of eggs laid on different plant structures for the flowering and reproductive crop stages in the no-choice oviposition experiment. Floral structures are summed up for clarity; they include bud initials, buds, flowers, and spent flowers. Bars represent means and error bars represent standard errors. Different letters indicate a difference according to Fisher’s LSD test.

Where moths laid their eggs differed among the crop stages, which differed in the availability of plant structures (Fig. 2; Supplementary Table S1). Because few eggs were laid in the vegetative treatment (Fig. 1; mean = 37), we only analyzed the distribution of eggs in the reproductive crop stage treatments (Fig. 2). How eggs were distributed among plant structures differed for both flowering (*F* = 18.18; *df* = 3,26; *P* < 0.001) and podding plants (*F* = 4.71; *df* = 3,17; *P* = 0.014); at flowering most *H. armigera* eggs were laid on floral structures, whereas at podding most eggs were laid on pods (Fig. 2).

**Fig. 3.**
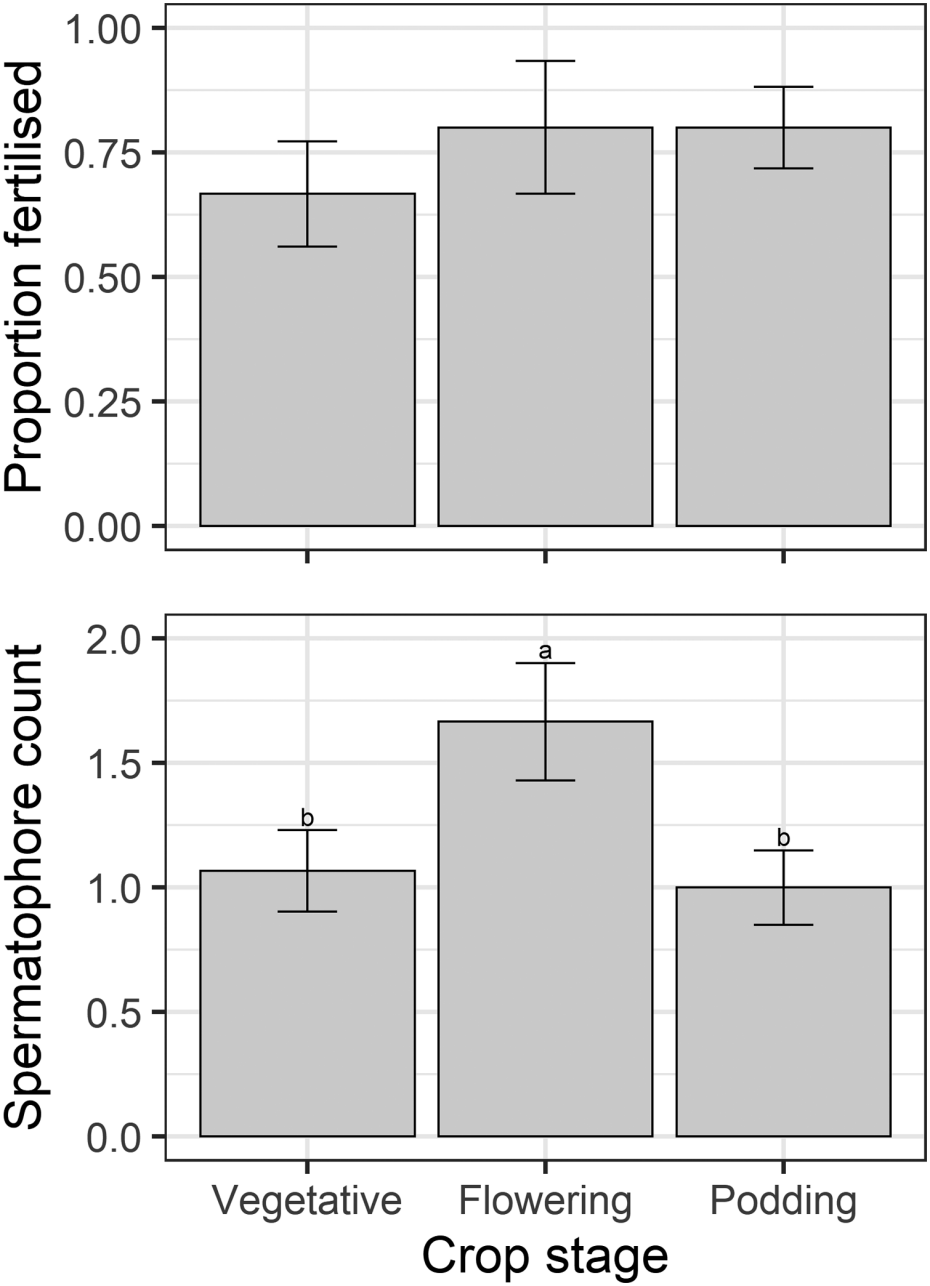
Female moth dissection data from the no-choice oviposition experiment—the proportion of moths fertilized per cage (*n* = 3 females per cage) and the average spermatophore count per female. Values are means ± standard errors. Different letters indicate differences in the means according to Fisher’s LSD test.

Of the 45 female *H. armigera* moths collected from the oviposition experiment (final 5 replicates only), 3 moths died in their cages (2 in a single flowering replicate and 1 in a vegetative replicate). There was no difference between the proportion of moths that were fertilized in different crop stages (*F* = 1; *df* = 2,8; *P* = 0.4096; Fig. 3), although there was a slight effect of replicate (*F* = 4; *df* = 4,8; *P* = 0.045). There was a significant effect of crop stage on the count of spermatophores per moth (*F* = 8.27; *df* = 2,8; *P* = 0.011; Fig. 3) along with replicate (*F* = 4.41; *df* = 4,8; *P* = 0.036), with moths in flowering cages having slightly more spermatophores (Fig. 3).

### Caged Early Instar Establishment—3 Days Duration

**Fig. 4.**
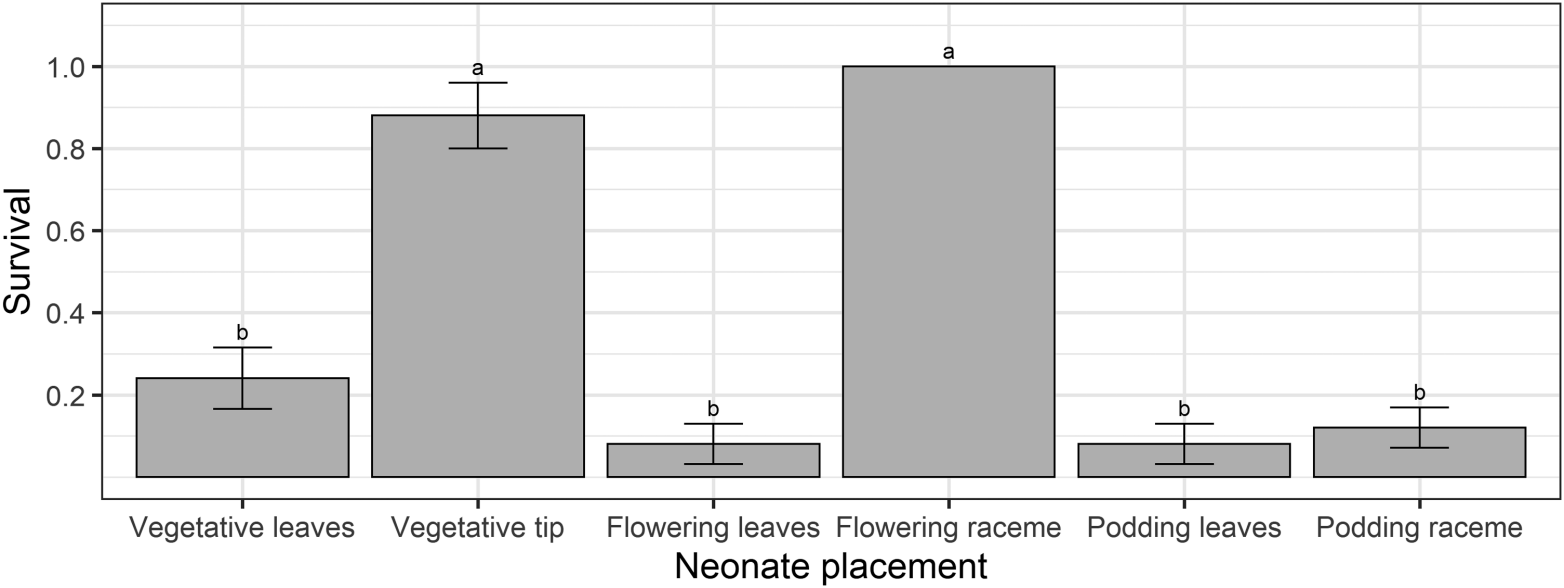
Larval survival when restricted to different locations on vegetative, flowering, and podding pigeon pea plants in the caged early instar establishment experiment. Bars represent means and error bars represent standard errors. Different letters indicate a difference according to Fisher’s LSD test. There is no error bar for the flowering raceme treatment because all replicates had 100% survival.

Early instar survival was significantly affected by crop stage × location treatments (*F* = 61.87; *df* = 5,20; *P* < 0.001). Larval survival was highest when released neonates were restricted to racemes of flowering plants (100%) and the growing tips of vegetative plants (88%) (Fig. 4). The small number of surviving larvae in most treatments precluded analysis of developmental data. However, at the time of assessment, all larvae in the flowering raceme and 78% of those in the vegetative tip treatment had reached the second instar.

Of the 150 *H. armigera* neonates placed on plants, 72 (48%) larvae were relocated after 3 days. Of these, 60 were alive, and 12 were cadavers. We relocated all larvae in the flowering raceme treatment and 88% of larvae in the vegetative tip experiment, but only 32%, 12%, 12%, and 44% from the vegetative leaves, flowering leaves, podding leaves, and podding raceme treatments, respectively.

### Uncaged Early Instar Establishment—3 Days Duration

**Fig. 5.**
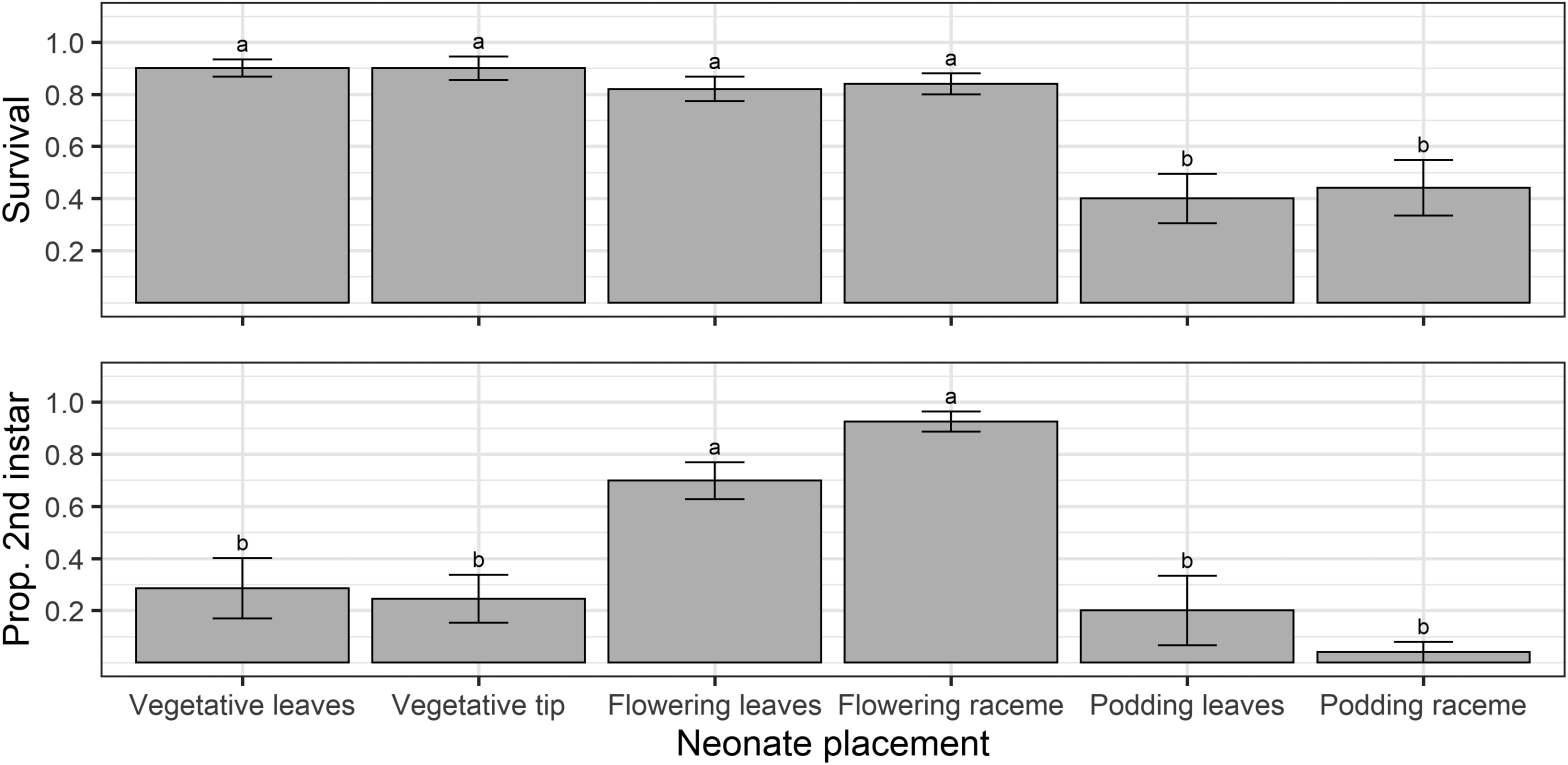
Larval survival and development from the uncaged early instar experiment. Values on the *y*-axis are mean proportions, and error bars represent the standard error of the mean. Different letters indicate a significant difference among treatments according to Fisher’s protected LSD test.

In the uncaged early instar establishment experiment, crop stage and plant location influenced larval survival (*F* = 11.68; *df* = 5,45; *P* < 0.001; Fig. 5). Larval survival was greatest on vegetative and flowering plants, regardless of what plant location neonates were released, and lowest on podding plants (Fig. 5). There was a strong treatment effect for larval development, with larvae on flowering plants developing the fastest (*F* = 15.31; *df* = 5,45; *P* < 0.001; Fig. 5).

Of the 300 *H. armigera* neonates placed on plants in the uncaged experiment, we were able to relocate 219 (73%) after 3 days. Of these, 215 were alive, and 4 were cadavers. We were only able to find 46% of the larvae placed on podding plants. On vegetative and flowering plants we were able to find 90% and 83% of larvae, respectively.

**Fig. 6.**
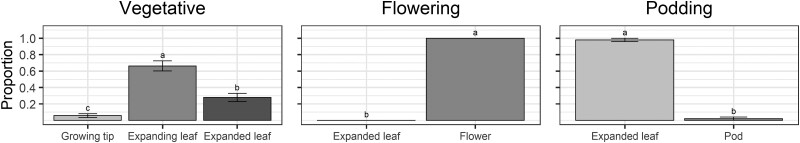
Larval distributions from the uncaged early instar experiment. Values on the *y*-axis are the mean proportions of larvae at plant locations after 3 days, and error bars represent the standard error of the mean. Different plant placement location treatments were pooled as placement location did not influence final larval distribution (Supplementary Fig. S1). Different letters indicate a significant difference in larval distribution within the crop stage treatments. Fisher’s protected LSD test was used to compare the distributions in the vegetative stage, and Kruskal–Wallis tests were used for the flowering and podding stages. Not all locations are present for all crop stages (i.e., vegetative plants lack flowers and pods; flowering plants lack expanding leaves, growing tips, and pods; and podding plants lack expanding leaves, growing tips, and flowers).

Data from larvae placed at different plant locations were pooled within crop stages as, for a given crop stage, larval distributions at day 3 were identical, irrespective of where they were originally placed on the plant (Supplementary Fig. S1). The distribution of larvae among plant structures differed for vegetative plants (*F* = 40.53; *df* = 3,56; *P* < 0.001; Fig. 6), flowering plants (*χ*^2^ = 39, *df* = 1, *P* < 0.001; Fig. 6), and podding plants (*χ*^2^ = 31.27, *df* = 1, *P* < 0.001; Fig. 6). On vegetative plants, most larvae were found on expanding leaves (66%), followed by expanded leaves (28%), with few larvae found at the plant growing tip (6%). On expanding leaves, 98% of larvae were found on the adaxial surface and only a single larva (2%) was found on the abaxial surface. On expanded leaves, however, only 15% were on the adaxial surface, and 85% were on the abaxial surface. In both flowering treatments, 100% of larvae were found in flowers. Most of these larvae (99%) were located inside flowers, and only a single larva was located outside the flower. In the flowering treatment, 6 second-instar larvae were found feeding on flowers, and the flowers that they were feeding on had all started to desiccate, and small expanding pods were emerging. In all 6 cases, these small pods had suffered feeding damage. In the podding treatments, most (98%) larvae were found on expanded leaves, and only a single larva was found in a large pod. Of the larvae on expanded leaves, most (93%) were found on the abaxial surface, and few (7%) were found on the adaxial surface.

### Unrestricted Larvae on Whole Plants—7 Days Duration

**Fig. 7.**
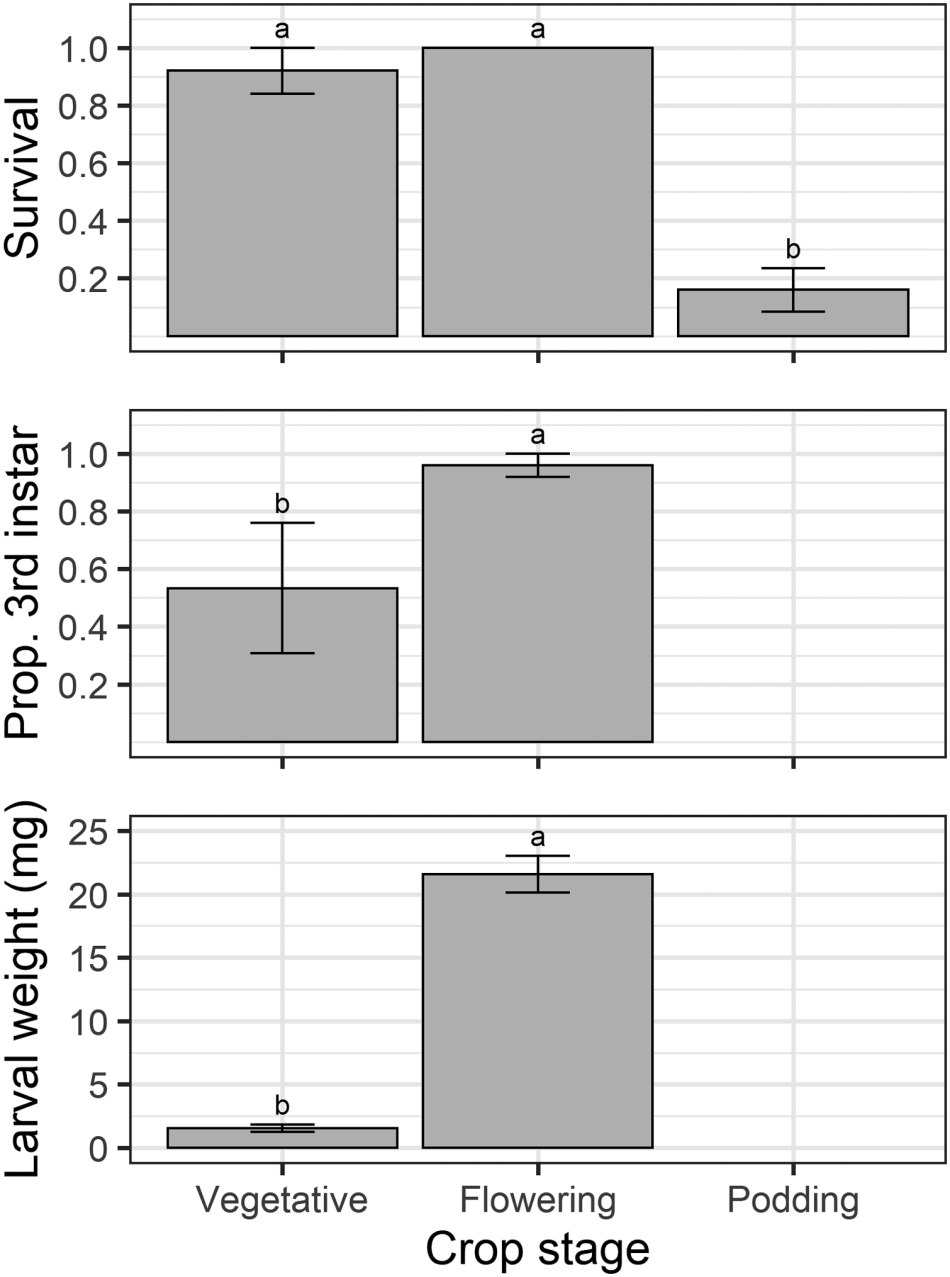
Larval performance measures (survival, development, and weight) from the 7 days unrestricted larvae experiment. Values on the *y*-axis are means and error bars represent the standard error of the mean. Different letters indicate a significant difference among treatments according to Fisher’s protected LSD test.

In the 7-day unrestricted larvae experiment, crop stage significantly influenced larval survival (*F* = 50.38; *df* = 2,8; *P* < 0.001), development (*F* = 8.2; *df* = 2,6; *P* = 0.019), and weight (*F* = 160.49; *df* = 2,6; *P* < 0.001) (Fig. 7). Larvae placed on flowering plants and vegetative plants were equally likely to survive; however larvae placed on flowering plants developed faster and weighed over 10 times more than those on vegetative plants (Fig. 7). All performance metrics were lowest on podding plants.

Of the 75 larvae placed on plants in the 7-day performance experiment, 52 (69%), were relocated, and all were alive. There was no difference in larval distribution among structures for vegetative plants (*F* = 1.77; *df* = 2,8; *P* = 0.23); 44% of larvae were on expanded leaves, 39% on expanding leaves, and 17% at the growing tip. For flowering plants, larvae were distributed nonrandomly among plant structures (*F* = 23.72; *df* = 2,12; *P* < 0.001), most larvae (88%) were on or inside a floral structure, and 12% were on emerging pods, but none were found on leaves. In the podding treatment, only 4 larvae were found alive, and all of them were on leaves. Given the small number of surviving larvae, the podding distribution data were not analyzed.

### Larval Survival on Plant Reproductive Structures—4 Days Duration

**Fig. 8.**
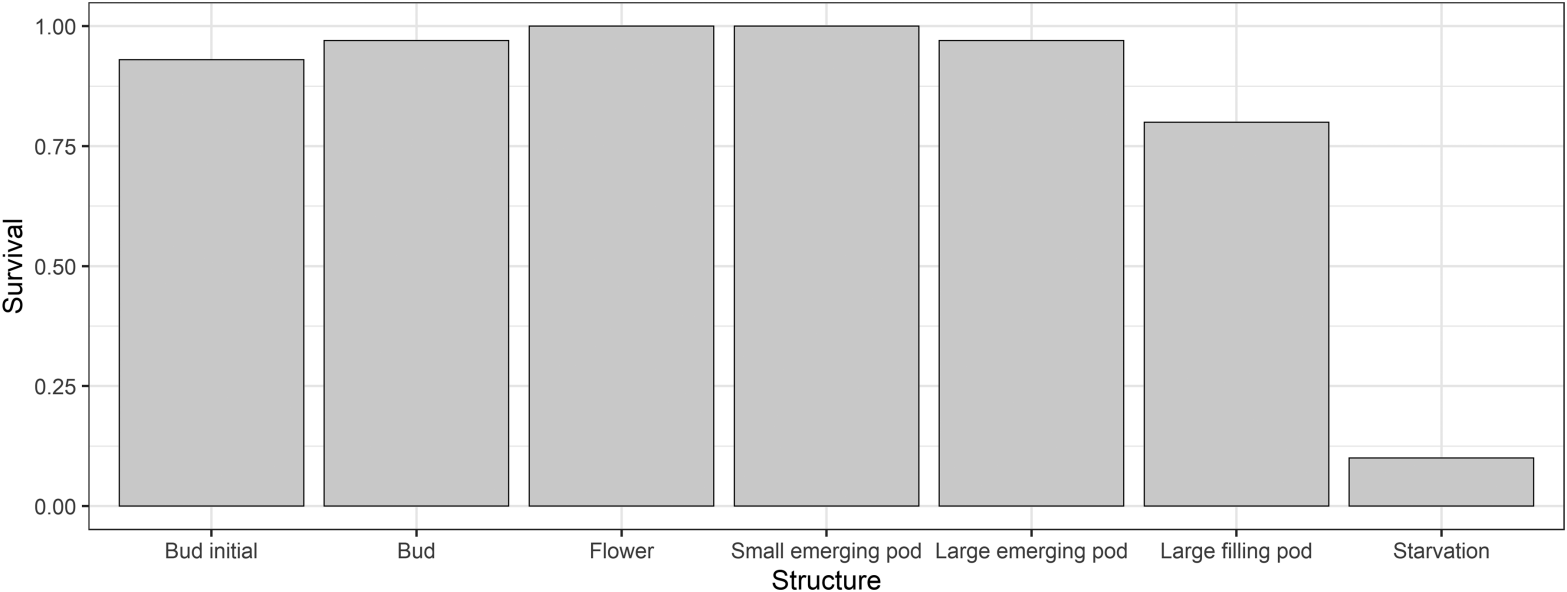
Proportion of larvae that survived after 96 h on different pigeon pea reproductive structures in the Petri dish assay, *n* = 30 replicates per treatment.

After 4 days, larval survival was strongly influenced by feeding treatment (*χ*^2^=132.15, *df* = 6, *P* < 0.001) (Fig. 8). Only 10% of larvae in the starvation treatment survived the 96h, indicating *H. armigera* larvae were able to feed and survive for at least 96 h on the various structures. We tried to identify the cause of any larval mortality, and in the large emerging pod treatment, the single dead larva was in pod exudate. Similarly, in the large filling pod treatment, 4 larvae were found dead in pod exudate. However, there were several larvae for which the cause of mortality could not be determined: 2 in the large filling pod treatment, 2 in the initial bud treatment, and 1 in the bud treatment.

## Discussion

We examined how pigeon pea crop stage influences *H. armigera* preference and performance to understand the crop’s susceptibility to its major pest. *Helicoverpa armigera* moths laid most eggs on reproductive stage plants (both flowering and podding) and laid very few eggs on vegetative plants. Larvae established best on vegetative and flowering plants and worst on podding plants. When assays were conducted for 7 days, substantial differences in larval performance (weight and development) were detected between vegetative and flowering plant treatments. Neonate *H. armigera* larvae can feed on most pigeon pea reproductive structures when restricted in no-choice assays, but larvae display a strong preference for flowers when placed on whole plants with these structures available.

We found that *H. armigera* females lay many eggs on flowering and podding pigeon pea plants but not on vegetative plants (Fig. 1), even though moth fertility was similar among treatments (Fig. 3). The proclivity of *H. armigera* females for ovipositing on flowering plants of other plant species has already been documented (Firempong and Zalucki 1990, Diongue et al. 2004, Liu et al. 2010) and there is some experimental evidence to suggest that floral volatiles play an important role in attracting female moths to pigeon pea (Rajapakse et al. 2006). Pigeon pea pods also produce volatiles (Borges et al. 2023) and, therefore, may attract female moths. Yet there is also evidence that pigeon pea leaf volatiles are attractive to *H. armigera* moths (Rembold and Tober 1985). Borges et al. (2023) showed that reproductive pigeon pea plants emit higher levels of monoterpenes (α-pinene, β-myrcene, limonene, (*E*)-ocimene, linalool) than vegetative plants, and these compounds elicit a response from *H. armigera* females in electroantennography (Rajapakse et al. 2006). However, there are also electrophysiologically active green leaf volatiles ((*2*E)-hexanal, (3*Z*)-hexenyl acetate, (3*Z*)-hexenyl-2-methylbutyrate) (Rajapakse et al. 2006). We suspect volatiles are an important prealighting cue for *H. armigera* oviposition on pigeon pea, and their lower quantity and different composition on vegetative plants may partly explain our oviposition results.

The substantial oviposition recorded on pods of podding pigeon pea plants (Fig. 2) was surprising, as larval establishment at these sites was poor (Figs. 4, 5, 7, and 8). Female *H. armigera* moths prefer to oviposit on “hairy” surfaces (Zalucki et al. 1986), and in our study system, we suspect trichomes are an important postalighting cue for oviposition. Pigeon pea pods and the calyxes of flowers and pods both have high levels of trichomes (Romeis et al. 1999a, Sharma et al. 2009, Volp et al. 2023), and *H. armigera* prefers to oviposit at these sites (Romeis et al. 1999b, Volp et al. 2023). It seems that *H. armigera* has evolved to detect plant trichomes as an ovipositional cue, potentially to maximize egg adhesion and survival. However, trichome-dense sites may not be favorable for neonate establishment, and larvae may disperse to locate an appropriate nearby feeding site (Potter et al. 2012). For instance, *H. armigera* lays many eggs on pigeon pea calyxes (trichome-dense sites), but neonates do not feed there; instead, they relocate inside flower petals (Volp et al. 2023). Trichomes on pigeon pea pods appear to act as a “sensory trap” (Jones and Agrawal 2019), i.e., female moths are attracted to trichomes although they do not provide an adaptive benefit to larvae because there is nowhere “optimal” for neonates to establish. However, the role of trichomes in promoting egg adhesion and, therefore, survival has not been explored fully, and there are likely trade-offs between the suitability of the site for oviposition and proximity to optimal feeding sites. This area warrants further work, as the prospect of eliminating plant traits that stimulate oviposition or which divert ovipositing moths to oviposition sites that are unfavorable for neonate establishment both represent exciting resistance tactics.

Our results obtained from the larval performance experiments do not align with the PPH. Despite oviposition being high on both flowering and podding plants, larval survival and performance were poor on podding pigeon peas. The poor larval performance on podding plants is due to the fact that the only feeding sites available were large filling pods or expanded leaves. Our Petri dish assay demonstrated that large filling pods are the pigeon pea reproductive structure least suitable for early instar larval survival (Fig. 8), likely due to the thick trichome-covered pod wall. Although *H. armigera* larvae can feed on pigeon pea leaves, these sites are suboptimal, and larvae will avoid leaves when provided a choice of food source (Sison and Shanower 1994, Johnson and Zalucki 2005, Rajapakse and Walter 2007, Volp et al. 2023). We detected cadavers in caged and uncaged 3 days duration experiments but not in the uncaged 7 days experiment. Most larvae from the podding plant treatments were missing, indicating larvae either: (i) died and desiccated (desiccated neonates being difficult to detect on whole plants), (ii) dispersed off plants by silking or crawling, or (iii) were cannibalized by other larvae. It is difficult to identify whether the low survival rate on podding plants was a result of mortality from plant traits, starvation due to the inability to feed at available sites, larvae dispersing off plants, or cannibalism. Even though *H. armigera* larvae are well-known cannibals, this behavior is rare for first instars and primarily manifests as older instars eating younger instars (Kakimoto et al. 2003). Therefore, we suspect most unaccounted larvae either died or dispersed. The high mortality in the caged early instar larvae experiment (Fig. 4), where larvae could not disperse, supports the explanation of larvae dying and desiccating rather than dispersal. The difficulty in disentangling actual mortality from larval dispersal is a major limitation in studies on early instar caterpillars (Zalucki et al. 2002).

In the larval experiments, we found that *H. armigera* larvae selected specific feeding sites when given a choice on whole plants. Early instar larvae prefer to feed at the adaxial side of expanding leaves when placed on vegetative plants, inside flowers when placed on flowering plants, and on the abaxial side of expanded leaves when placed on podding plants (Fig. 6). Regardless of placement location, larvae relocated to their “preferred” feeding sites (Supplementary Fig. S1). There has been substantial work examining the movement of early instar *H. armigera* larvae, documenting that neonates move up towards the apex of plants and prefer to feed inside plant structures (Johnson and Zalucki 2005, Perkins et al. 2008, 2009, 2010, Cribb et al. 2010). Our results closely align with this body of work. In the vegetative treatments, larvae were mostly located “inside” the unfurling leaves at the plant apex, and in the flowering treatments, larvae were inside flowers, which are also at the top of the plant. However, in the podding treatments, larvae were predominately found on expanded leaves. The larval distribution on podding plants indicates that they avoid pods and instead prefer to feed on suboptimal structures (i.e., expanded leaves), plant parts that they typically avoided in the vegetative and flowering treatments.

We conducted the no-choice Petri dish assay to provide greater clarity around the larval distributions we observed in our performance experiments on whole plants. Surprisingly, we found larvae could survive (for 96 h at least) on all pigeon pea reproductive structures we provided to them (Fig. 8). Interestingly, most larvae on large filling pods survived, even though they strongly avoided these structures when placed on podding plants. We suspect it is worth investigating if larvae can survive and develop on these suboptimal structures if forced to feed on them for a longer period. The larval performance experiments demonstrate that (i) *H. armigera* neonates are surprisingly adept at feeding on different plant structures if restricted in Petri dish assays, (ii) unrestricted larvae on plants make important foraging decisions to select where they feed, and therefore (iii) it is important for experimenters to consider where caterpillars “choose” to feed rather than choosing their feeding sites for them when evaluating their larval survival/ performance on host plants.

In the current study, we placed neonate larvae on plants as they are the most susceptible larval stage to mortality in general (Zalucki et al. 2002) and for *H. armigera* (Kyi et al. 1991, Titmarsh 1992). However, to better understand the *H. armigera*-pigeon pea system, we need to consider how *H. armigera* larvae change their feeding behavior as they develop. Ontogenetic changes in feeding behavior have been documented in *H. armigera* feeding on both vegetative mungbean (Johnson and Zalucki 2007) and artificial diet in Petri dishes (Wang et al. 2019). A study on the closely related *Helicoverpa zea* documented the ability of fourth instars to survive when feeding on soybean pods, whereas second instars could only survive on leaves and flowers (Suits et al. 2017). Future work should investigate how the feeding behavior of *H. armigera* larvae on pigeon pea changes as larvae develop.

Elsewhere, we have advocated that researchers trying to develop *H. armigera*-resistant pigeon pea need to properly understand *H. armigera*-pigeon pea insect–plant interactions (Volp et al. 2023). In this study, we further elucidate aspects of the study system. We have shown that the susceptibility of pigeon pea to *H. armigera* is strongly tied to the flowering crop stage, as well as the availability of flowers for oviposition sites for moths and establishment sites for susceptible neonate caterpillars. We suspect future work exploring the interaction between pigeon pea phenology and *H. armigera* infestation will prove useful. For instance, can varieties be developed with more synchronous (interplant and intraplant) and shorter flowering windows, which will limit the pest infestation period? Additionally, what floral traits are available in current pigeon pea germplasm to decrease *H. armigera* oviposition and larval establishment? Investigating these questions will help develop management strategies for this key pest of pigeon pea.

## Supplementary Material

toae050_suppl_Supplementary_Table_S1

toae050_suppl_Supplementary_Figure_S1

toae050_suppl_Supplementary_Figure_Legend
