# Supplementary material for: Pigeon pea crop stage strongly influences plant susceptibility to Helicoverpa armigera (Lepidoptera: Noctuidae)
Source: J Econ Entomol. 2024 Apr 2;117(3):973–81. doi: 10.1093/jee/toae050 (PMC11163456; doi:10.1093/jee/toae050)
Supplement: toae050_suppl_Supplementary_Table_S1 [file toae050_suppl_supplementary_table_s1.docx]

**Table S1:** Plant morphometric data from the oviposition experiment. Values are means ± standard errors. Different letters indicate a significant difference according to Fisher’s LSD test. Significant differences were detected among crop stages for

plant height (F=36.07, df=2,30; P<0.001), mainstem node count (F=68.54; df=2,30; P<0.001), trifoliate leaves (F=114.7; df=2,30; P<0.001), bud initials (F=91.73, df=2,30; P<0.001), buds (F=85.17; df=2,30; p<0.001), flowers (F=152.1; df=2,30; P<0.001), spent flowers (F=374.5; df=2,30; P<0.001), small expanding pods (F=24.08; df=2,30; P<0.001), and large filling pods (F=314.3, df-2,30; P<0.001). There were no large expanding pods on any plants.

| **Crop stage** | **Plant height (mm)** | **Mainstem nodes** | **Trifoliate leaves** | **Bud initials** | **Buds** | **Flowers** | **Spent flowers** | **Small expanding pods** | **Large filling pods** |
| --- | --- | --- | --- | --- | --- | --- | --- | --- | --- |
| **Vegetative** | 439±26b | 11.6±0.9b | 9.6±0.8b | 0b | 0b | 0b | 0b | 0b | 0b |
| **Flowering** | 658±22a | 19.7±0.5a | 40.0±1.5a | 56.2±3.1a | 23.4±2.5a | 26.6±2.2a | 27.0±1.4a | 5.5±1.1a | 0b |
| **Podding** | 652±12a | 20.4±0.3a | 32.9±1.3a | 10.5±4.4b | 0b | 0b | 0b | 0b | 32.8±1.8a |
