# Supplementary figures and images for: Pigeon pea crop stage strongly influences plant susceptibility to Helicoverpa armigera (Lepidoptera: Noctuidae)
Source: J Econ Entomol. 2024 Apr 2;117(3):973–81. doi: 10.1093/jee/toae050 (PMC11163456; doi:10.1093/jee/toae050)

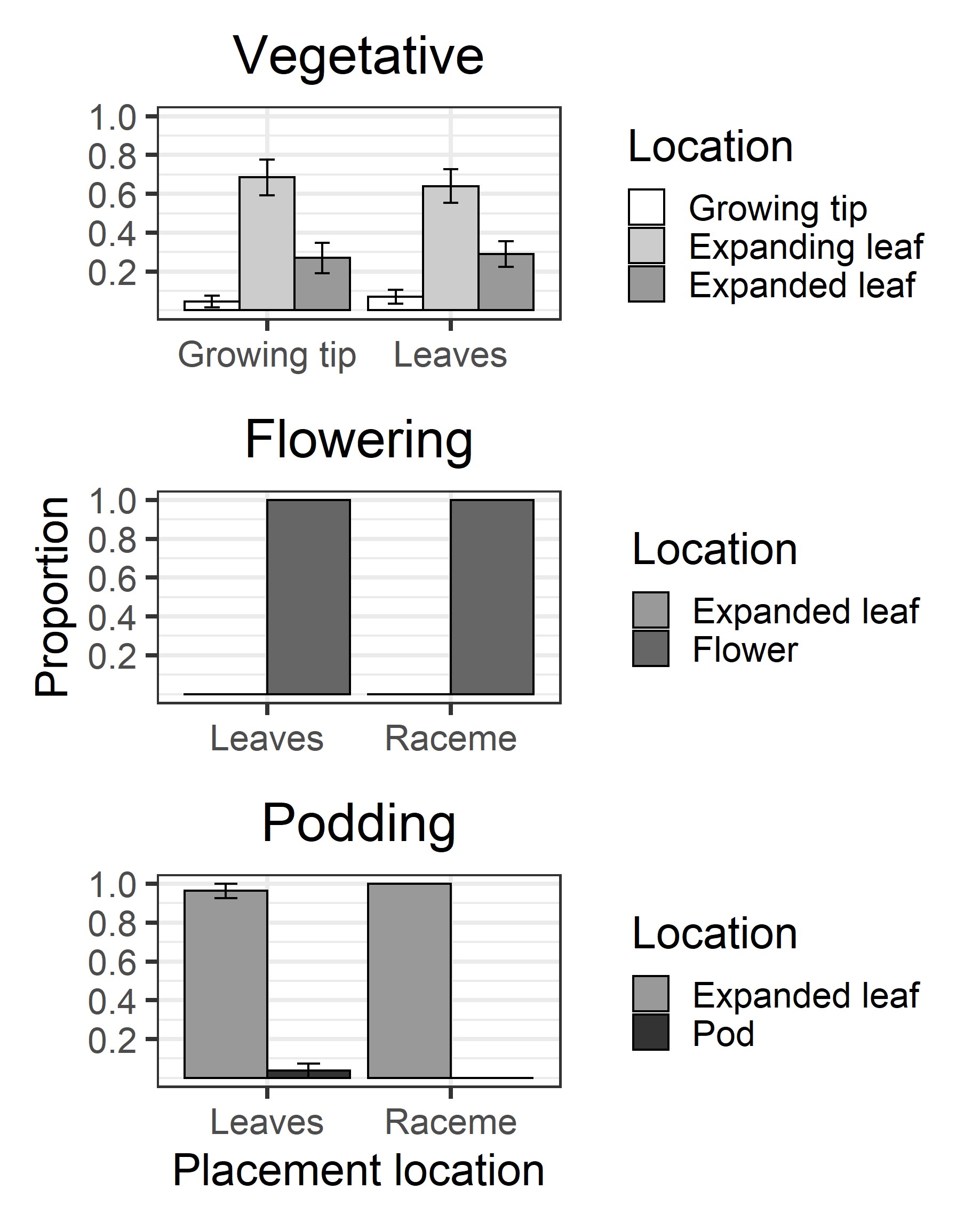

Supplement: toae050_suppl_Supplementary_Figure_S1 [file toae050_suppl_supplementary_figure_s1.jpeg]
