# Supplementary material for: Pigeon pea crop stage strongly influences plant susceptibility to Helicoverpa armigera (Lepidoptera: Noctuidae)
Source: J Econ Entomol. 2024 Apr 2;117(3):973–81. doi: 10.1093/jee/toae050 (PMC11163456; doi:10.1093/jee/toae050)
Supplement: toae050_suppl_Supplementary_Figure_Legend [file toae050_suppl_supplementary_figure_legend.docx]

**Fig. S1:** Distribution of larvae in the uncaged larval establishment experiment after 3d, different crop stages displayed in different panes. Bars represent average proportion of larvae found in a location per replicate, error bars are standard errors.
